# CBX4 promotes the proliferation and metastasis via regulating BMI‐1 in lung cancer

**DOI:** 10.1111/jcmm.14771

**Published:** 2019-11-13

**Authors:** Changpeng Hu, Qian Zhang, Qin Tang, Huyue Zhou, Wuyi Liu, Jingbin Huang, Yali Liu, Qin Wang, Jing Zhang, Min Zhou, Fangfang Sheng, Wenjing Lai, Jing Tian, Guobing Li, Rong Zhang

**Affiliations:** ^1^ Department of Pharmacy The Second Affiliated Hospital Army Medical University Chongqing China; ^2^ Department of Teaching Support Army Medical University Chongqing China

**Keywords:** B cell‐specific Moloney murine leukaemia virus integration site 1, Chromobox 4, lung cancer, metastasis, proliferation

## Abstract

Proliferation and metastasis are significantly malignant characteristics of human lung cancer, but the underlying molecular mechanisms are poorly understood. Chromobox 4 (CBX4), a member of the Polycomb group (PcG) family of epigenetic regulatory factors, enhances cellular proliferation and promotes cancer cell migration. However, the effect of CBX4 in the progression of lung cancer is not fully understood. We found that CBX4 is highly expressed in lung tumours compared with adjacent normal tissues. Overexpression of CBX4 significantly promotes cell proliferation and migration in human lung cancer cell lines. The knockdown of CBX4 obviously suppresses the cell growth and migration of human lung cancer cells in vitro. Also, the proliferation and metastasis in vivo are blocked by CBX4 knockdown. Furthermore, CBX4 knockdown effectively arrests cell cycle at the G0/G1 phase through suppressing the expression of CDK2 and Cyclin E and decreases the formation of filopodia through suppressing MMP2, MMP9 and CXCR4. Additionally, CBX4 promotes proliferation and metastasis via regulating the expression of BMI‐1 which is a significant regulator of proliferation and migration in lung cancer cells. Taken together, these data suggest that CBX4 is not only a novel prognostic marker but also may be a potential therapeutic target in lung cancer.

## 1 INTRODUCTION

Lung cancer is one of the most threatening malignancies and has the fastest‐growing incidence and high death rate.^1^ In recent years, the morbidity and mortality of lung cancer are significantly increased. In all malignancies, the morbidity and mortality of lung cancer are the highest in men and ranks second in women. Although the mortality rate has been controlled by surgical techniques and chemotherapy, the survival rate of patients with lung cancer is still very low.^2^ As proliferation and metastasis are significant characteristics of lung cancer to prognosis, a better elucidation of the processes that control proliferation and metastasis in lung cancer may be providing new possible therapeutic strategies for lung cancer treatments.^3^, ^4^

Polycomb repressive complex 1 (PRC1) is a member of polycomb group (PcG) family, and PRC1 is a kind of target gene with the function of transcriptional suppressor of chromatin modification and regulation. These are abnormal proteins of epigenetic regulation and play an important role in the occurrence and metastasis in tumour.^5^ PRC1 contains BMI‐1, RING1, HPH and HPC proteins.^6^, ^7^ BMI‐1 (B cell‐specific Moloney murine leukaemia virus integration site 1) is a polycomb ring finger oncogene which plays a crucial role in cell growth, metastasis and stem cell self‐renewal.^8^, ^9^, ^10^, ^11^, ^12^, ^13^ It has been reported that BMI‐1 is a potential therapeutic target for glioma.^14^ Clinical studies revealed that BMI‐1 expression was negatively correlated with survival of patient with colon cancer.^15^ It has recently reported that CBX4 is an important upstream regulator of BMI‐1, controlling the sumoylation status of BMI‐1 and regulating BMI‐1 recruitment to sites of DNA damage in mammalian cells.^16^

Chromobox family has five members including CBX2, CBX4, CBX6, CBX7 and CBX8, which is a subgroup of protein in the PcG family, and they have distinct biological functions in different tissues.^17^ For example, CBX8 has been reported to be a growth‐promoting protein in leukemogenesis and bladder cancer,^18^, ^19^ whereas it acts as an oncogene in colorectal carcinoma.^20^ CBX7 is a tumour suppressor that shows low expression in human cancers and recruits HDAC2 to the CCNE1 promoter to suppress CCNE1 expression in lung cancer.^21^ CBX4 (a SUMO E3 ligase, known as HPC2) is a relatively specific PcG protein involved in tumour occurrence and cell cycle regulation. Recently, evidence has revealed that CBX4 is a cell cycle inhibitor gene of proliferative activity in the epithelium.^22^ Under normoxic conditions, CBX4 acts as an up‐regulated protein with a pro‐tumour effect by activating the HIF‐1α signalling pathway in osteosarcoma.^23^ In addition, CBX4 is a new therapeutic target for hepatocellular carcinoma, as high expression of this protein leads to poor overall survival.^17^, ^24^ In general, researchers proved that CBX4 plays an important role in the occurrence and development of tumours. However, the mechanism underlying the interactive functions of CBX4 and BMI‐1 has not yet been fully documented.

In this study, we firstly demonstrated that CBX4 regulated proliferation and migration by regulating the expression of BMI‐1 in lung cancer cells. Notably, CBX4 knockdown inhibited the abilities of proliferation and migration in lung cancer cells, thereby decreasing the expression of BMI‐1. Furthermore, BMI‐1 overexpression could reverse the inhibition caused by CBX4 in proliferation and migration, but it could not reverse for the expression of CBX4. Our study provides a novel insight into the proliferation and migration of CBX4 and suggests that knockdown of CBX4 reduces the abilities of proliferation and metastasis via BMI‐1 in lung cancer.

## 2 MATERIALS AND METHODS

### 2.1 Tissues

Sixty formalin‐fixed and paraffin‐embedded specimens of lung cancer tumours and paired adjacent normal tissues were collected from 30 patients at Southwest Hospital, Chongqing, China (from June 2011 to June 2013). The study was approved by the Ethics Committee of the Institutional Review Board of the Army Medical University. Written informed consent was obtained from all patients. All samples were registered by a case number in the database with no patient names or personal information. The following demographic data were recorded: sex, age, tumour size, clinical stage and lymph node metastasis. Pathological diagnosis was done according to the criteria described by American Joint Committee on Cancer (AJCC).

### 2.2 Antibodies

The following antibodies were used in our study. Anti‐CBX4 (bs‐17376R, 1:400) was purchased from Bioss. Anti‐BMI‐1 (YM0072, 1:1000), anti‐MMP2 (YT2798, 1:1000), anti‐MMP9 (YT5357, 1:1000) and anti‐CXCR4 (YM3546, 1:500) were purchased from ImmunoWay Biotechnology. Anti‐CDK2 (sc‐163, 1:1000), anti‐CDK5 (sc‐6247, 1:1000), anti‐Cyclin E (sc‐481, 1:1000), anti‐cdc2 (sc‐54, 1:1000), anti‐Cyclin B1 (sc‐245, 1:500) and anti‐P53 (sc‐126, 1:1000) were obtained from Santa Cruz Biotechnology (USA). Anti‐GAPDH (AG019‐1, 1:10000) was purchased from Beyotime Biotechnology. Anti‐Ki67 (ab45580, 1:1000) was from Abcam.

### 2.3 Cell culture

NCI‐H1299, PC9, NCI‐H460, A549 and MES‐1 cells were purchased from the American Type Culture Collection (ATCC), which authenticates cell lines with short tandem repeat profiling and monitoring cell morphology. Cells were maintained in Dulbecco's Modified Eagle's Medium (DMEM) containing 10% FBS and 1% penicillin/streptomycin at 37°C in 5% CO_2_. Cells were digested and passaged after every two days.

### 2.4 Lentiviral infection and plasmid transfer

For the knocking down of CBX4, CBX4 shRNA (5′‐GACACCAGTAACCTTGGTAT‐3′) was synthesized and cloned into PGMLV‐SC6 plasmid. Lentivirus vectors encoding shCBX4 were constructed (Genemeditech). NCI‐H460 and A549 cells were infected with lentivirus at 20 multiplicity of infection (MOI) and grown under 8 μg/mL puromycin (Invitrogen, A1113803) selection to obtain the stable cell lines. For the overexpression of CBX4, the human wt‐CBX4 plasmid was purchased from Sino Biological Inc (# HG29716‐UT). The human wt‐BMI‐1 plasmid was a gift from Jesse Boehm & William Hahn & David Root (Addgene plasmid # 82 194; http://n2t.net/addgene:82194; RRID: Addgene_82194).^25^ The plasmids were transfected into NCI‐H460 and A549 cells with Lipofectamine 3000 (Invitrogen, L3000015) according to the manufacturer's instructions. After 48 hours, Western blot analysis was used to confirm the efficiency.

### 2.5 Cell viability (CCK8) assay

Cells were seeded in 96‐well plates (500 cells per well) and were treated as indicated for 1 to 5 days. About 10 μL of CCK8 (C0039; Beyotime Biotechnology) solution was added per well and incubated for 2 to 4 hours before measuring with a microplate reader at 450 nm. The cell viabilities were normalized to the control group.

### 2.6 Western blot

Cells and tissues were lysed with RIPA lysis buffer (P0013B; Beyotime Biotechnology) and detected with Enhanced BCA Protein Assay Reagent (P0010; Beyotime Biotechnology). Each lysate was separated on 10%‐12% SDS‐PAGE gels and then transferred onto PVDF membranes (162‐0177; Bio‐Rad). The membranes were blocked with 5% nonfat dried milk for 2 hours and subsequently incubated with primary antibodies overnight at 4°C. After washing three times with TBST, the membranes were incubated with a secondary antibody for 2 hours, and then the bands were visualized with the enhanced chemiluminescence kit (170‐5061; Bio‐Rad). The densitometric analysis was measured by Quantity One software (Bio‐Rad).

### 2.7 Clonogenicity assay

Cells were seeded in 5‐cm dishes (100 cells per dish) in DMEM with 10% FBS. Then the cells were cultured for 2‐3 weeks and the media in all the dishes were changed after every week. After the clones were visible in all groups, clones were fixed with 4% paraformaldehyde for 15 minutes and stained with crystal violet (3%) for 10 minutes. The images were acquired with a digital camera.

### 2.8 Cell cycle

Cells were seeded in 6‐well plates and cultured overnight. Cells were harvested and washed twice with ice‐cold PBS, suspended in 250 μL of ice‐cold PBS, and then slowly added to 750 μL of 100% ice‐cold ethanol. After 8 hours of fixation, the cells were washed with PBS and incubated with RNase (50 μg/mL) and propidium iodide (PI) (50 μg/mL, Thermo Fisher) for 30 minutes. Next, the cell cycle was performed by Flow Cytometry System (BD Accuri C6) and the relative ratios of G0/G1, S and G2/M phases were analysed by ModFit LT 5.0 software (Verity Software House).

### 2.9 Immunofluorescence

Cells were seeded on glass coverslips and cultured in 24‐well plates. After 24 hours, cells were fixed with 1 mL of 75% alcohol for 15 minutes, permeated with 0.1% Triton X‐100 for 10 minutes and further incubated in blocking buffer (5% FBS) for 30 minutes. Cells were then incubated with anti‐Ki67 (ab15580, 1:200; Abcam) for overnight at 4°C, and then finally incubated with Alexa fluor 488 anti‐mouse secondary antibody (Santa Cruz Biotechnology) for 1 hour. For staining the cytoskeleton, cells were incubated with Alexa fluor 488 Phalloidin (8878S; CST), and then counterstained with 4′, 6‐diamidino‐2‐phenylindole (DAPI, Sigma‐Aldrich). After counterstaining with 0.1 mg/mL DAPI for 5 minutes, cells were visualized by a laser‐scanning confocal microscope (LSM780NLO; Zeiss). Quantification of Ki67‐positive puncta was measured by Zeiss LSM Image Examiner software.

### 2.10 Cell monolayer scratching assay

NCI‐H460 and A549 cells were seeded in 6‐well plates with DMEM containing 10% FBS until 95% confluence. A 1‐mL pipette tip was used to scratch the bottom of the plates in a straight line. After washing with PBS, the wounds were monitored at 0 and 48 hours using a Cell Imaging System (ZEISS). The migratory ability of the cells was measured as the distance of the wound recovered at 48 hours compared with 0 hour.

### 2.11 Transwell assay

The lower chambers of Transwell (3422; Corning) were filled with 600 μL of DMEM medium containing 30% FBS. The upper chambers were seeded with a total of 5000 cells in 200 μL of serum‐free DMEM. After 48 hours of incubation, the membranes were fixed with 75% alcohol for 15 minutes. The cells on the upper surface of the membrane were wiped with a cotton swab carefully, and the cells on the other side of the membrane were stained with 3% crystal violet solution (Beyotime Biotechnology). Then each well was filled with PBS and photographed under a microscope (CKX41; Olympus). The number of invading cells was counted in three randomly selected fields.

### 2.12 In vivo proliferation assays

Male 4‐ to 5‐week‐old BABL/c nude mice were purchased from the Laboratory Animal Center of the Army Medical University and housed in a pathogen‐free environment. All animal procedures were approved by the Army Medical University Animal Committee. Lung cancer cells (1 × 10^6^ cells in 0.2 mL Matrigel and DMEM medium (1:1, v/v)) were injected subcutaneously into the lower back of the mice. Tumour growth was observed every day and tumour volume was evaluated every week. After 5 weeks, the mice were killed and the tumour was removed. The tumour volume was evaluated using the following formula: tumour volume = (width/2)^2^ × (length/2).

### 2.13 In vivo metastasis assays

For the metastasis model, the spleens of mice were injected with a total of 1 × 10^6^ cells stably. Five weeks after injection, the mice were killed and the livers were removed for pathological examination. Then different transverse sections of metastasis were prepared for H&E staining and immunohistochemistry analysis, and metastatic nodules were counted in a double‐blind manner.

### 2.14 Immunohistochemistry (IHC) and IHC Evaluation

The tissues of human beings and mice were subjected to IHC staining to detect the expression levels of CBX4, BMI‐1 and Ki67. Immunohistochemistry was performed as described.^26^ The following antibodies were used: anti‐CBX4 (1:300) or anti‐BMI‐1 (1:300) and anti‐Ki67 (1:200). The expression of CBX4 was evaluated by the immunoreactive score (IRS) described by Remmele and Stegner.^27^ In brief, value of 0, 1, 2, 3 or 4 was assigned to 0%, 1%‐25%, 26%‐50%, 51%‐75% and 76%‐100% of positive cells, respectively, while 0, 1, 2 and 3 scores were used to represent the staining intensity of positive cells which were graded as negative, weak, moderate and strong, respectively.. The product of the extent score and the intensity was the immunoreactive score (IRS). The range of IRS was from 1 to 12, which were graded as follows: ‐ (IRS 0), + (IRS 1‐4), ++ (IRS 5‐8) and +++ (IRS 9‐12).

### 2.15 Statistical analysis

Data were presented as mean ± SD. Statistical analysis was performed using GraphPad Prism version 6.0 (GraphPad Software Inc). Chi‑squared tests were performed to evaluate the significance of the associations between CBX4 and clinicopathological parameters. The statistical evaluation was performed by Student's *t*‐test. A one‐way ANOVA was used for multiple comparisons followed by the Bonferroni method. The results were considered significant at ^*^ *P* < .05, ^**^ *P* < .01 and ^***^ *P* < .001.

## 3 RESULTS

### 3.1 CBX4 expression was associated with human lung cancer tissues and cells

**Figure 1:**
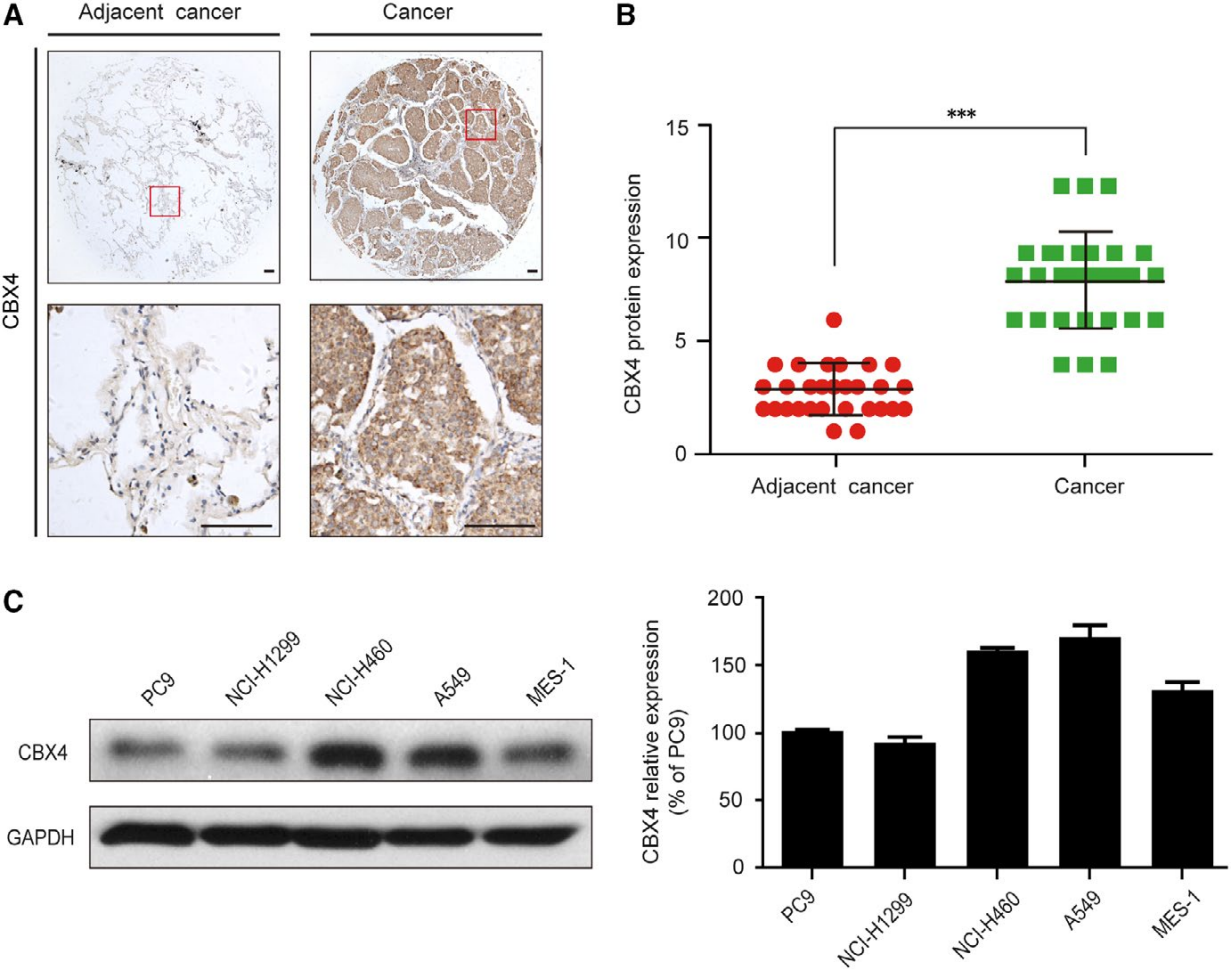
The relative expression of CBX4 was elevated in human lung cancer tissues and cells. A, Representative immunohistochemical images of CBX4 in human lung cancer and adjacent cancer tissues. Scale bars: 100 μm. B, The CBX4 expression levels in lung cancer and adjacent cancer tissues (n = 30). C, The expression level of CBX4 was analysed by Western blot in lung cancer cells (PC9, NCI‐H1299, NCI‐H460, A549 and MES‐1 cells) and the histograms show the CBX4 relative expression. Densitometric measurements were analysed using Quantity One software. The protein expression levels were normalized to those of the vehicle control (100%). Data are presented as means ± SD (^***^ *P* < .001)

First, we measured the CBX4 expression levels in human lung cancer tissues (30 cases) and adjacent normal tissues by immunohistochemistry. Our results showed that the expression of CBX4 in human lung cancer tissues was significantly higher than in the adjacent normal tissues (^***^ *P* < .001, Figure 1A,B). In addition, we analysed the correlation between CBX4 expression levels and different clinicopathological factors in lung cancer. Results showed that the expression of CBX4 showed a significant positive correlation with tumour size (*P* = .0015), clinical stage (^***^ *P* < .001) and lymph node metastasis (^***^ *P* < .001), but no significant associations were found between CBX4 expression and gender (*P* > .05) and age (*P* > .05) (Table 1). Meanwhile, we measured the CBX4 expression levels in human lung cancer cell lines PC9, NCI‐H1299, NCI‐H460, A549 and MES‐1. The results indicated that the expression of CBX4 was high in NCI‐H460 and A549 cells which were selected for conducting the following study (Figure 1C). Taken together, these results demonstrate that CBX4 is overexpressed in human lung cancer cells and contributes to increased tumour growth and metastasis, suggesting that CBX4 is a positive lung cancer regulator. Monitoring the expression level of CBX4 in lung cancer tissues may provide new diagnostic, prognostic and therapeutic strategies for clinical lung cancer treatment.

**Table 1:** Correlation between CBX4 expression and clinicopathological factors in lung cancer patients

Clinicopathological factors	High expression, (n = 20)	Low expression, (n = 10)	*P*‐value
Gender			
Male	8	3	.0665
Female	12	7	
Age			
≥60	11	6	.2672
＜60	9	4	
Tumour size			
≥3	15	5	.0015**
＜3	5	5	
Clinacal stage			
Ⅰ	4	4	<.001***
Ⅱ‐Ⅲ	16	9	
Lymph node metastasis			
Yes	16	7	<.001***
No	4	3	

** *P* < .01;

*** *P* < .001.

### 3.2 CBX4 overexpression promotes cell proliferation and migration of NCI‐H460 and A549 cells in vitro

**Figure 2:**
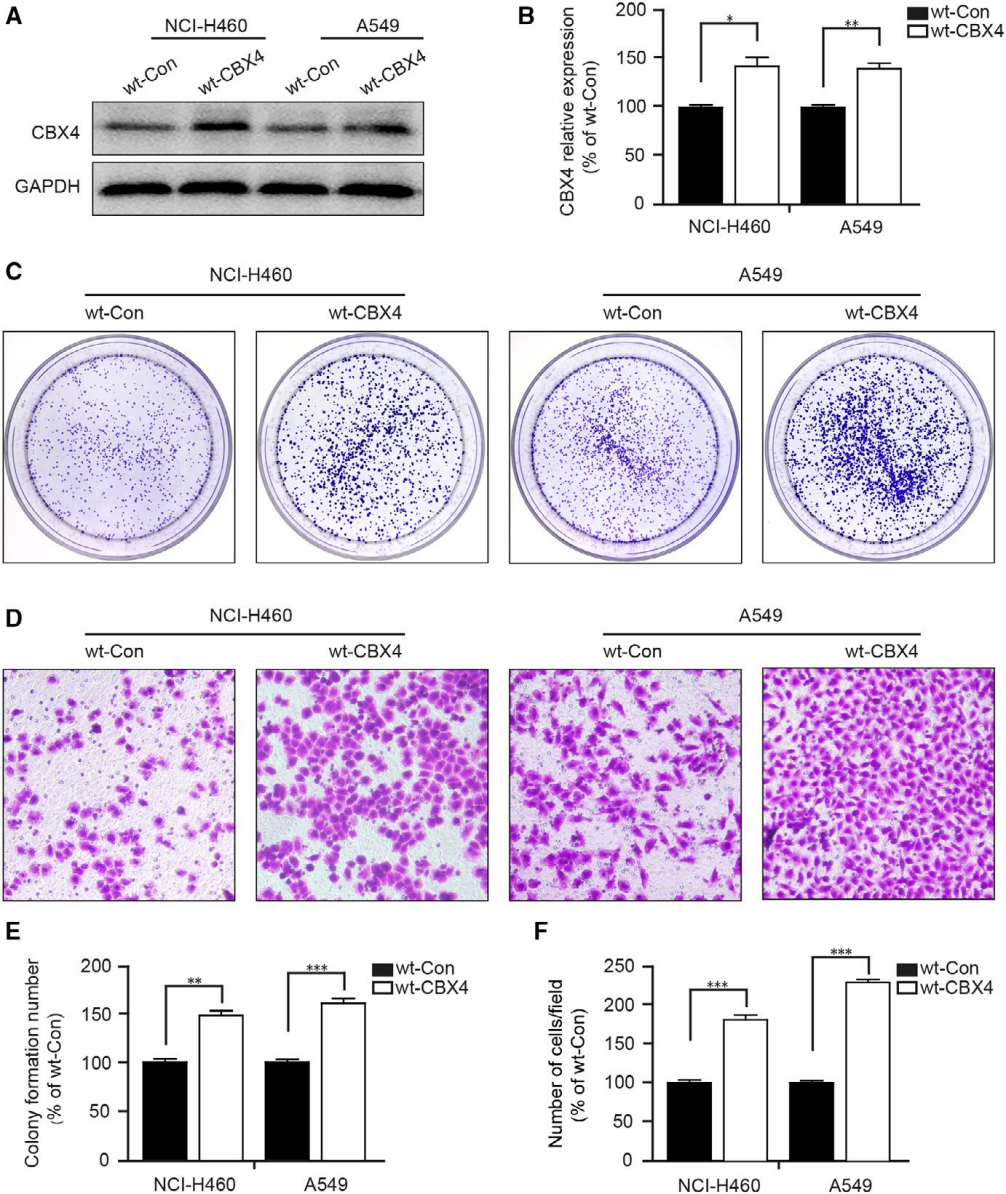
CBX4 overexpression promotes cell proliferation and migration of NCI‐H460 and A549 cells. NCI‐H460 and A549 cells were transfected with wt‐CBX4 plasmid. A, The expression levels of CBX4 was measured by Western blotting and (B) analysed by Quantity One software. C, In colony formation assay, cells were fixed and stained with crystal violet after culturing for 2 weeks and (E) counted the stained cells. D, Cells were seeded in the upper chambers of the transwells for 48 h and stained with crystal violet. F, The migrated cells were counted in representative images and the histograms show the ratio of the migrated cells to the control from three independent fields. Scale bars: 200 μm. All data were presented as mean ± SD (^*^ *P* < .05, ^**^ *P* < .01, ^***^ *P* < .001)

In view of the overexpression of CBX4 in lung cancer tissues, we assessed the effects of CBX4 overexpression on cell proliferation and migration of lung cancer. CBX4 expression was significantly increased in both NCI‐H460 and A549 cells transfected with wild‐type cofilin (wt‐CBX4) plasmids (^*^ *P* < .05, ^**^ *P* < .01, Figure 2A,B). In addition, our colony formation assay showed that CBX4 overexpression markedly increased the colony formation ability of NCI‐H460 and A549 cells compared with the wt‐Con groups (^**^ *P* < .01, ^***^ *P* < .001, Figure 2C,E). Moreover, our transwell assay revealed that CBX4 overexpression promoted cell migration in NCI‐H460 and A549 cells (^***^ *P* < .001, Figure 2D,F). These results indicated that CBX4 overexpression promotes cell proliferation and migration in lung cancer cell lines.

### 3.3 CBX4 knockdown decreases cell proliferation of lung cancer cells in vitro

**Figure 3:**
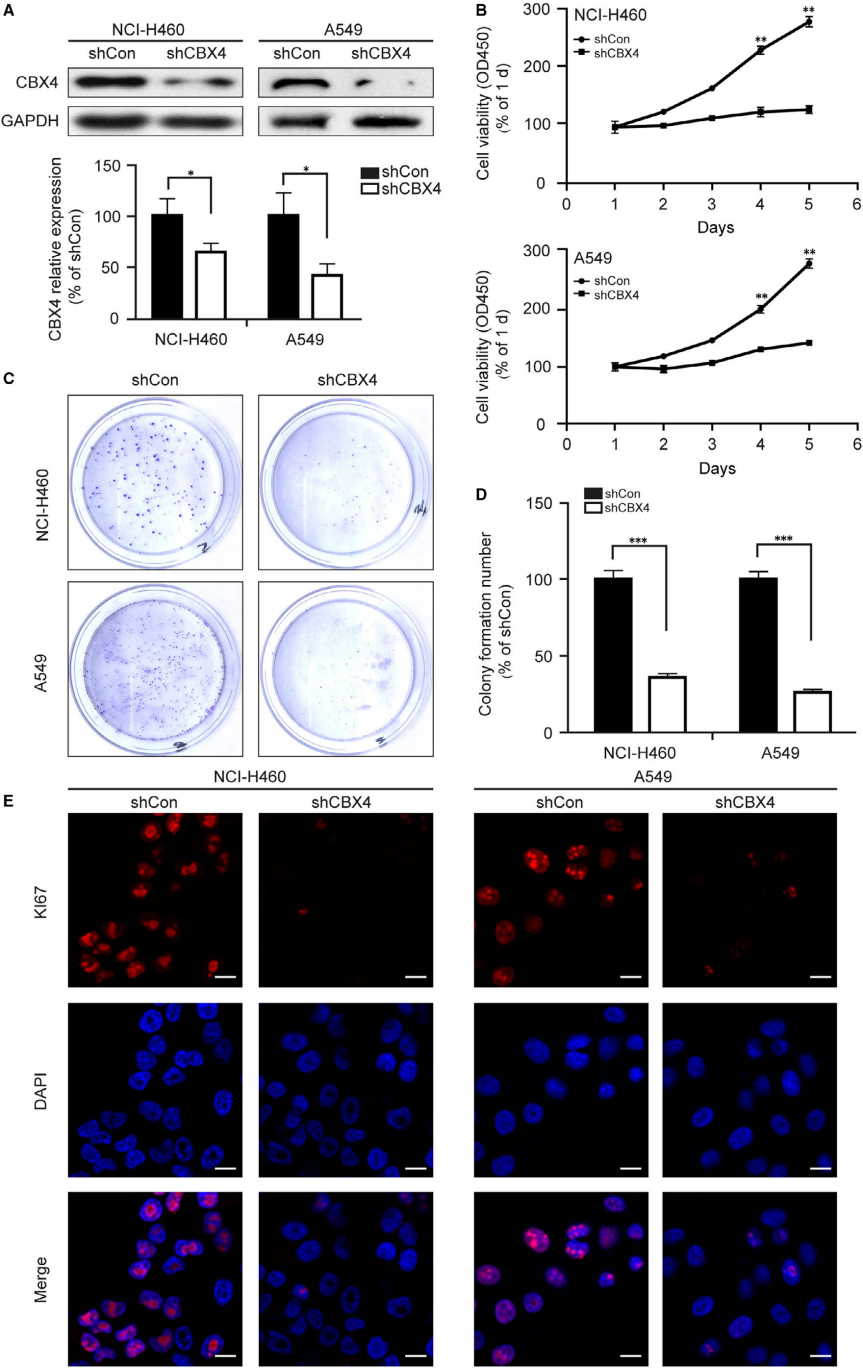
CBX4 promotes the proliferation of lung cancer cells. NCI‐H460 and A549 cells were infected with shCon or shCBX4 lentivirus. A, The knockdown efficiency of shCBX4 was measured by Western blotting. B, Cell viability was measured by a microplate reader at 450 nm using CCK8 assay at the indicated time. (C and D) In colony formation assay, NCI‐H460 and A549 cells were fixed and stained with crystal violet after culturing for 2 wk, and the stained cells were counted in three independent experiments. E, NCI‐H460 and A549 cells were immunostained with Ki67 (red) and DAPI (blue). Photos were obtained by confocal microscopy and measured by Zeiss LSM Image Examiner software. Scale bars: 20 μm. All data were presented as mean ± SD (^*^ *P* < .05, ^**^ *P* < .01, ^***^ *P* < .001)

Subsequently, we evaluated the effect of the knocking down of CBX4 expression in NCI‐H460 and A549 cells by Western blotting (^*^ *P* < .05, Figure 3A). CCK8 assays indicated that CBX4 knockdown diminished the growth of NCI‐H460 and A549 cells (^**^ *P* < .01, Figure 3B). In addition, our colony formation assay showed that CBX4 knockdown significantly decreased the colony formation in NCI‐H460 and A549 cells (^***^ *P* < .001, Figure 3C,D). Moreover, we measured the expression of Ki67 which was a marker indicating the proliferation of cells. Our immunofluorescence data showed that knockdown of CBX4 markedly decreased the expression of Ki67 in NCI‐H460 and A549 cells (Figure 3E). The findings suggest that CBX4 is a promoter for lung cancer cell proliferation.

### 3.4 G0/G1‐arrested cells were susceptible to CBX4 knockdown

**Figure 4:**
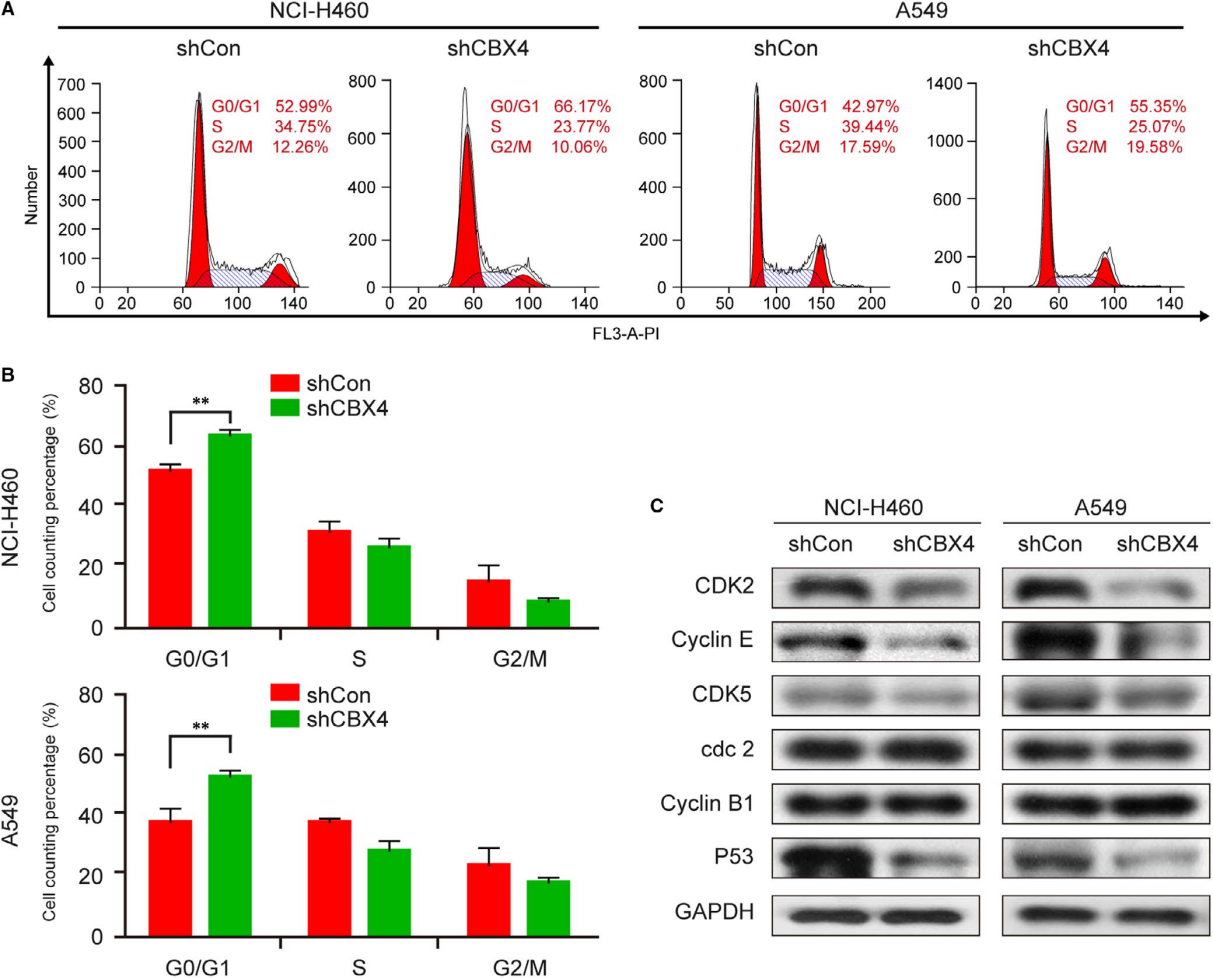
Knockdown of CBX4 arrested cell cycle at the G0/G1 phase in lung cancer cells. (A and B) Stable expression of shCon or shCBX4 in NCI‐H460 and A549 cells was analysed by flow cytometer. Cell cycle distribution (%) was measured using the ModFit LT 5.0 software. C, The expression of cell cycle‐related proteins CDK2, Cyclin E, cdc2, Cyclin B1 and P53 was analysed by Western blotting. GAPDH was used as loading control. Data were presented as mean ± SD (^**^ *P* < .01)

Cell growth is influenced by cell cycle regulation which plays a key factor in proliferation.^28^, ^29^ To determine whether knockdown of CBX4 affects the cell cycle, a flow cytometry assay was employed. Knockdown of CBX4 prominently increased the percentage of cells at the G0/G1 phase in both NCI‐H460 and A549 cells (^**^ *P* < .01, Figure 4A,B). Furthermore, we detected the expression of cell cycle regulatory molecules using Western blotting. The knocking down of CBX4 significantly reduced the expression of G0/G1 phase regulators CDK2, Cyclin E and CDK5, whereas knockdown of CBX4 had no effect on the expression of G2/M phase regulators cdc2 and Cyclin B1 (Figure 4C). Meanwhile, CBX4 knockdown also decreased the expression of P53, which was an important molecule for the G1/S transition in the cell cycle. Taken together, these data prove that CBX4 is a positive regulator of growth in the lung cancer cells by regulating the cell cycle, and that knockdown of CBX4 induces cell cycle arrest at the G0/G1 phase.

### 3.5 CBX4 knockdown decreases cell migration and invasion of lung cancer cells in vitro

**Figure 5:**
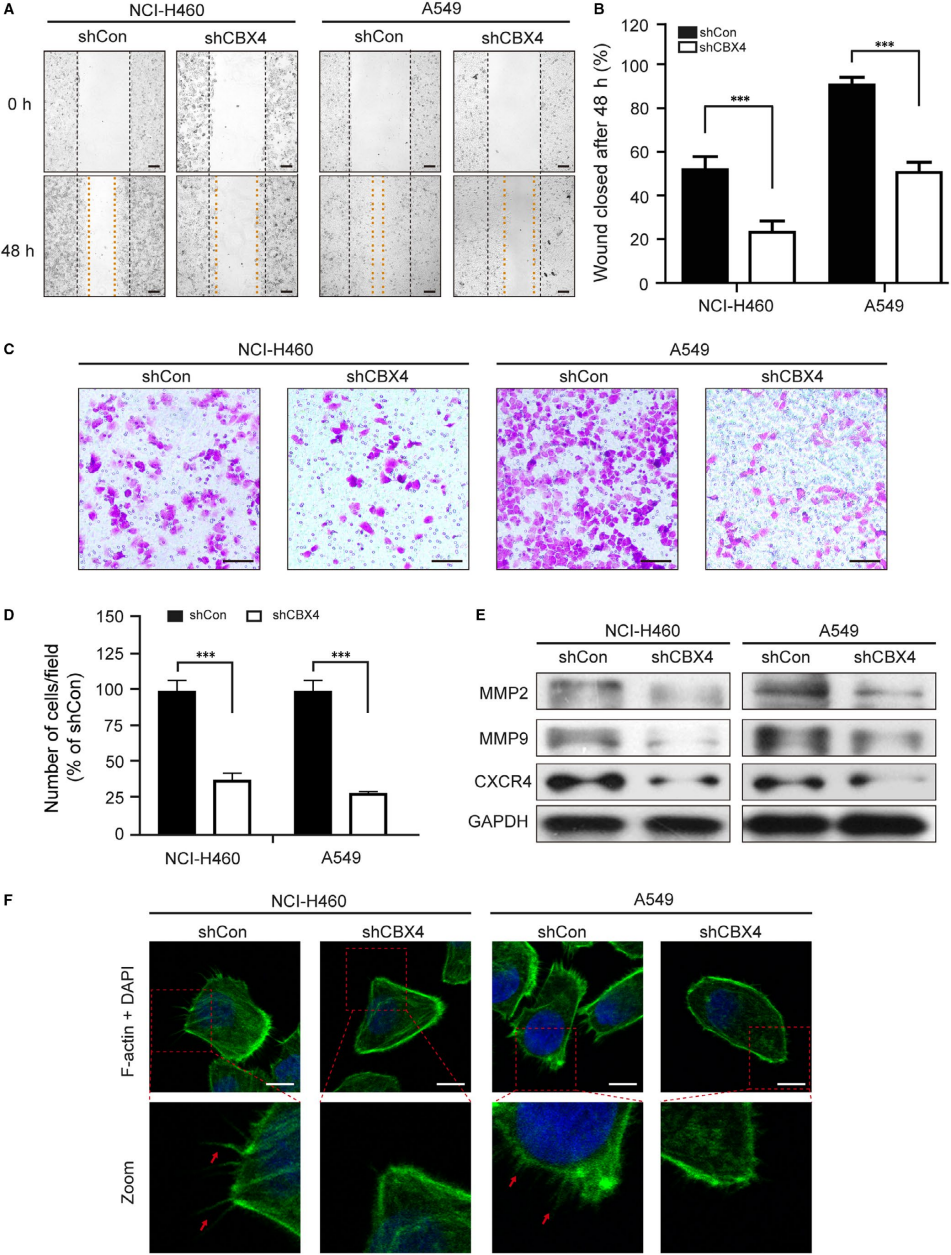
The knockdown of CBX4 reduces the migratory ability in NCI‐H460 and A549 cells. (A and B) Cells were seeded in 6‐well plates and the scratch migration assay was performed to test the migratory potential of NCI‐H460 and A549 cells after infecting with shCon or shCBX4 lentivirus. The ratios of the wound closure were measured at three different sites in each image. Scale bars: 200 μm. C, Cells were seeded and stained with crystal violet using transwell assay. D, The histograms show the ratio of the migrated cells to the control from three independent fields. Scale bars: 200 μm. E, Cells were seeded on the coverslips and immunostained with F‐actin (green) and DAPI (blue). Scale bars: 10 μm. F, The relative proteins of migration (MMP2, MMP9, CXCR4) and loading control were analysed by Western blot. All data were presented as mean ± SD (^***^ *P* < .001)

To further investigate the effect of CBX4 on the migration and invasion in lung cancer cells, a scratch migration assay and transwell assay were performed. As shown in Figure 5A,B, the scratch migration assay indicated that knockdown of CBX4 significantly decreased the wound closure of NCI‐H460 and A549 cells (^***^ *P* < .001, Figure 5A,B), suggesting knockdown of CBX4 inhibits cell migration. Similarly, transwell assay also demonstrated that CBX4 knockdown inhibited cell invasion in NCI‐H460 and A549 cells (^***^ *P* < .001, Figure 5C,D). In addition, our Western blot analysis showed that knockdown of CBX4 decreased the expression of migration‐related proteins MMP2, MMP9 and CXCR4 compared to that in control cells (Figure 5E). To further illustrate the role of CBX4 in migration, we observed the filopodia formation of lung cancer cells.^30^, ^31^ As shown in Figure 5F, the length and quantity of filopodium in CBX4‐knockdown cells were markedly decreased compared to control cells (Figure 5F). Together, these results prove that the knockdown of CBX4 decreased the migratory and invasive abilities of lung cancer cells in vitro.

### 3.6 CBX4 regulates proliferation and migration of NCI‐H460 and A549 cell via BMI‐1

**Figure 6:**
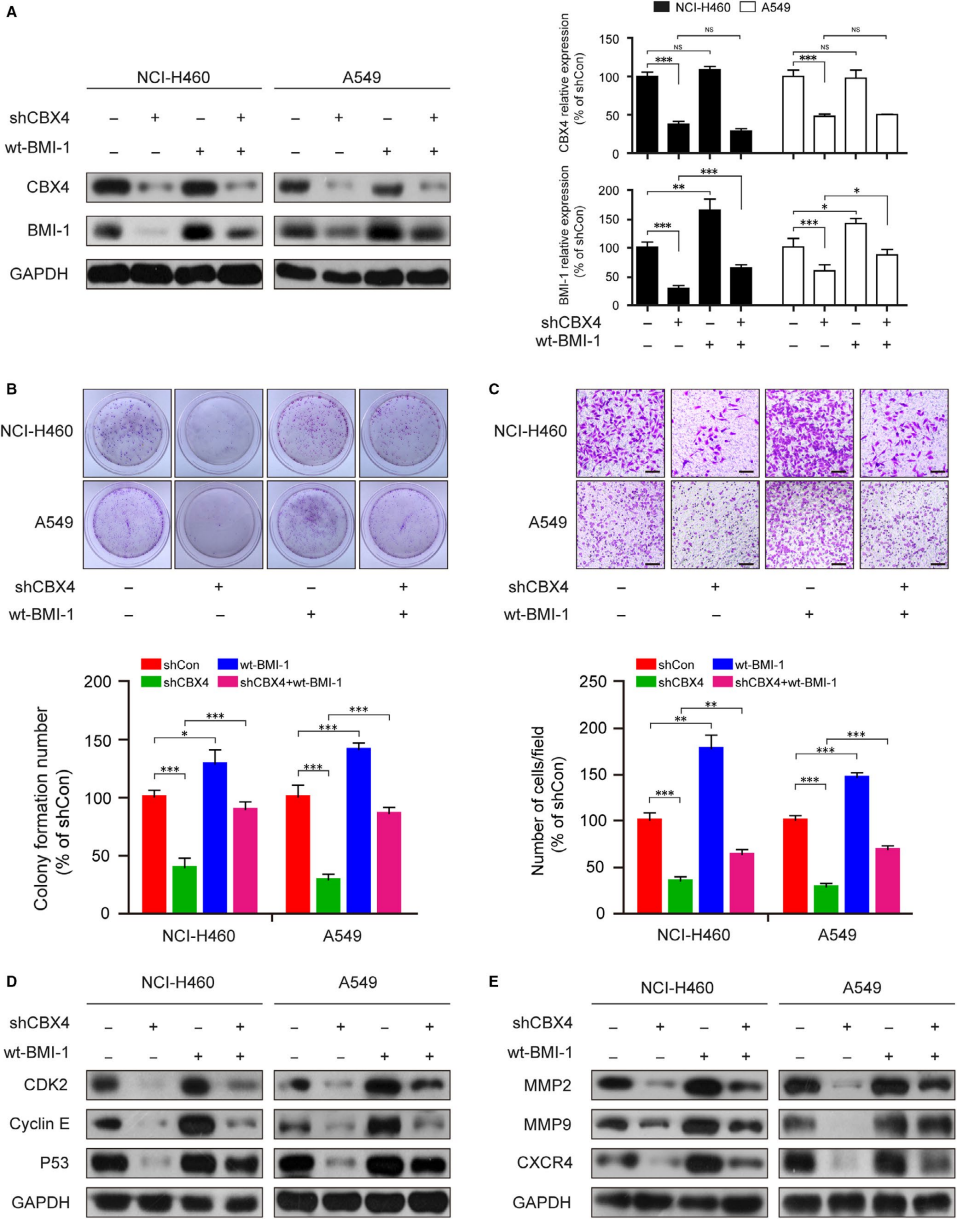
The knockdown of CBX4 attenuates the abilities of proliferation and migration in lung cancer cells via decreasing BMI‐1 expression. NCI‐H460 and A549 cells were infected with shCBX4 lentivirus or transfected wt‐BMI‐1 plasmid. A, The expression levels of CBX4 and BMI‐1 were measured by Western blotting and analysed by Quantity One software. B, In colony formation assay, cells were fixed and stained with crystal violet after culturing for 2 wk and the stained cells were counted. C, Cells were seeded and stained with crystal violet using transwell assay. Scale bars: 200 μm. (D and E) The relative expression of proteins of proliferation (CDK2, Cyclin E, P53) and migration (MMP2, MMP9, CXCR4) was analysed by Western blotting. GAPDH was used as an internal control. All data were presented as mean ± SD (^*^ *P* < .05, ^**^ *P* < .01, ^***^ *P* < .001)

It has been reported that BMI‐1 was involved in regulating the proliferation and metastasis of the tumour.^9^, ^12^ As reported, CBX4 is an important upstream regulator of BMI‐1, regulating the sumoylation status of BMI‐1 and BMI‐1 recruitment to sites of DNA damage in mammalian cells. To determine whether CBX4 exhibits a positive effect on cell proliferation and metastasis via regulating the expression of BMI‐1, NCI‐H460 and A549 cells stably expressing shCBX4 and/or wt‐BMI‐1 were used. First, we examined the expression of CBX4 and BMI‐1 in NCI‐H460 and A549 cells. CBX4 knockdown obviously reduced the expression of CBX4 (^***^ *P* < .001 vs control) and BMI‐1 (^***^ *P* < .001 vs control), while overexpression of BMI‐1 markedly increased the expression of BMI‐1 (*P < .05 and **P < .01 vs control) and also attenuated the CBX4 knockdown‐reduced BMI‐1 expression (^*^ *P* < .05 and ^***^ *P* < .001, shCBX4 vs shCBX4 + wt‐BMI‐1). However, overexpression of BMI‐1 did not reverse CBX4 expression (*P* > .05) (Figure 6A). In addition, colony formation assay and transwell assay were performed to examine the functional effects of overexpression of BMI‐1 combined without or with shCBX4 on cell proliferation and migration. CBX4 knockdown markedly reduced the colony formation ability, whereas BMI‐1 overexpression could reverse the phenomenon (Figure 6B). Consistent with these findings, transwell assay revealed that overexpression of BMI‐1 attenuated shCBX4‐inhibited cell invasion (Figure 6C). Moreover, we measured the expression of proliferation‐ and migration‐relating proteins such as CDK2, Cyclin E, P53, MMP2, MMP9 and CXCR4 by Western blotting. CBX4 knockdown obviously decreased cell cycle‐related proteins CDK2, Cyclin E and P53 levels, whereas BMI‐1 overexpression could reverse the decrease of these proteins induced by shCBX4 (Figure 6D). Similar results were also found in cell migration‐related proteins, where overexpression of BMI reversed shCBX4‐induced down‐regulation of MMP2, MMP9 and CXCR4 (Figure 6E). Taken together, these results suggest that the expression of BMI‐1 is regulated by CBX4, and that BMI‐1 overexpression can effectively reverse the function caused by knocking down of CBX4 in lung cancer cells.

### 3.7 Knocking down of CBX4 reduces the tumourigenic ability and metastasis of lung cancer in vivo

**Figure 7:**
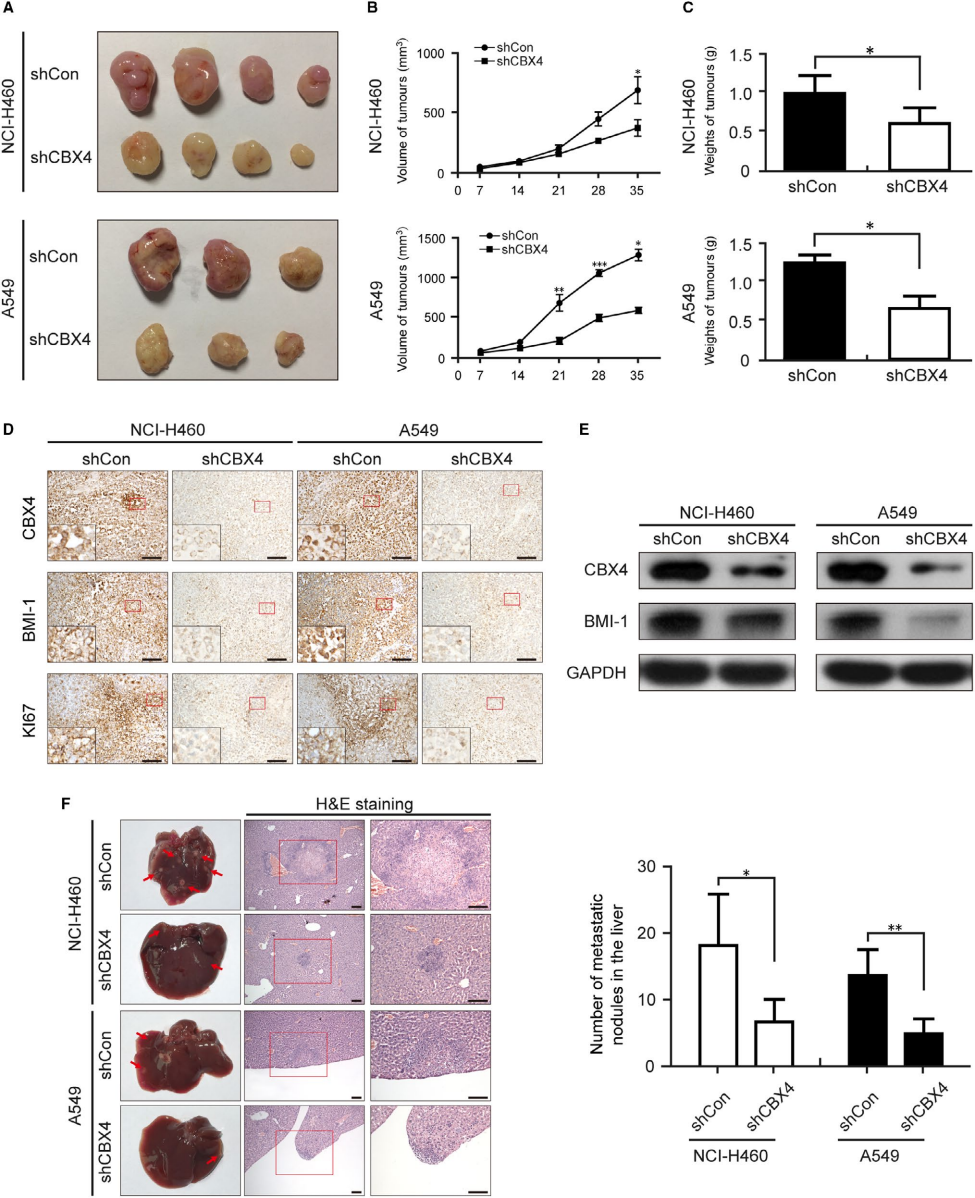
CBX4 knockdown reduces the proliferation and metastasis of lung cancer in vivo. NCI‐H460 and A549 cells were injected into the corresponding part depending on the different models. A, After 5 wk, the nodular tumour was harvested and (B) the volumes of the tumour were measured every other week. The tumours in the CBX4‐silencing groups were obviously smaller than in the control group. C, The weights of the xenografts were measured at the end of experiment. D, Images for CBX4, BMI‐1 and Ki67 staining are shown for the xenografts. Scale bars: 100 μm. E, The expressions of CBX4 and BMI‐1 in xenograft tumours were analysed by Western blotting. F, Representative livers and H&E staining of the metastatic nodules in the livers were from the indicated groups. Metastatic nodules are pointed out by arrows. Statistical results of the metastatic nodules are presented as mean ± SD (^*^ *P* < .05, ^**^ *P* < .01, ^***^ *P* < .001)

We then evaluated the effect of CBX4 knockdown on the tumourigenic ability of NCI‐H460 and A549 cells using a xenograft model in immunodeficient nude mice. shCon and shCBX4 cells were injected into nude mice subcutaneously which were fed for 5 weeks. The tumour volumes and weights of mice with CBX4 knockdown were lower than those observed in the shCon group (^*^ *P* < .05, Figure 7A‐C), suggesting that knockdown of CBX4 inhibits tumour growth in lung cancer xenograft mouse model. To determine whether CBX4 knockdown reduced the ability of proliferation and the expression of CBX4 and BMI‐1, immunohistochemical analyses were performed. As shown in Figure 7D, knockdown of CBX4 markedly decreased the expression of CBX4, BMI‐1 and Ki67 in vivo compared with shCon group (Figure 7D). Similar results were found by Western blotting, which showed that knockdown of CBX4 suppressed the expression of BMI‐1 in tumour tissues (Figure 7E). These findings indicate that knockdown of CBX4 inhibits NCI‐H460 and A549 xenograft growth via inhibiting BMI‐1.

To determine whether CBX4 knockdown reduced the metastasis of lung cancer in vivo, we then developed hepatic metastasis models by injecting cells into the spleens of nude mice for 8‐10 weeks. At the termination of the experiment, livers were harvested and evaluated by H&E staining. As shown in Figure 7F, CBX4 knockdown resulted in the formation of smaller and fewer metastatic liver nodules (Figure 7F), compared with that observed in control groups. Overall, these results indicate that CBX4 knockdown inhibits the growth and metastasis of lung cancer cells in vivo.

## 4 DISCUSSION

Recurrence and metastasis are the most common lethal factors after curative resection in lung cancer.^32^ It is necessary to investigate the underlying mechanisms of cell proliferation and metastasis of lung cancer. CBX4 is a specific PcG protein involved in tumour occurrence and cell cycle regulation. CBX4 expression varies in different types of cancer and has diverse biological functions. It is a cell cycle promoting gene with proliferative activity and is found in the epithelium; this protein is found to be up‐regulated and exhibits pro‐tumour effect by activating the HIF‐1α signalling pathway in osteosarcoma.^16^, ^22^, ^23^ In our study, we demonstrated that the expression of CBX4 in lung tumour tissues was significantly higher compared with adjacent cancer tissues (Figure 1A,B). Meanwhile, higher CBX4 expression was positively correlated with tumour size, clinical stage and lymph node metastasis (Table 1). In addition, we found that CBX4 overexpression promoted lung cancer progression by significantly enhancing the proliferation and migration of NCI‐H460 and A549 cells. (Figure 2) The results suggest that CBX4 was significantly overexpressed in lung cancer and may be a potential therapeutic target.

Based on the results of clinical tissues and CBX4 overexpression, we boldly conjecture that knockdown of CBX4 may reduce the proliferation and migration of lung cancer cells. Our in vitro studies demonstrated that CBX4 knockdown reduced the cell viability and colony formation (Figure 3B‐D). Ki67 is a nuclear protein and is a cell proliferation marker with wide application in a clinical setting for cancer diagnosis.^33^, ^34^ Our data indicated that the knockdown of CBX4 decreases the expression of Ki67 compared with the control group (Figure 3E). It has been reported that Ki67 exists in all active phases of the cell cycle (G1, S, G2), but is absent in the resting cells (G0 phase), suggesting that CBX4 knockdown may induce cell cycle arrest at the G0 phase.^35^ Consistent with this notion, our flow cytometry assay proved that knockdown of CBX4 significantly increased the percentage of cells at the G0/G1 phase in both NCI‐H460 and A549 cells (Figure 4A,B). P53 binds several genes including microRNA, miR‐34a and WAF1/CIP1 encoding for P21 which binds to G1‐S/CDK complexes (molecules important for the G1/S transition in the cell cycle) inhibiting their activity.^29^ Cyclin E binds to CDK2/CDK5 and plays a critical part in cell cycle, and low expression of Cyclin E and CDK2 has been reported to inhibit G1/S transition. Cdc2 and CyclinB1 are reportedly G2/M phase regulators, which are found to be highly expressed in G2/M phases of cell cycle. 28, 36 In this report, we found that knockdown of CBX4 decreased the expression of P53, CDK2, and Cyclin E, whereas no changes were observed in the expression of cdc2 and Cyclin B1, indicating that knockdown of CBX4 arrested cell cycle at the G0/G1 phase via regulating the expression of P53, CDK2 and Cyclin E (Figure 4C). Taken together, these results suggest that CBX4 is a positive regulator of the growth in lung cancer cells by regulating cell cycle, and knockdown of CBX4 induces cell cycle arrest at the G0/G1 phase.

It has been reported that metastasis is the major problem in lung cancer and the CBX family could play a significant role in controlling the migration of cancer cells.^21^ MMP2 and MMP9 are capable of degrading type IV collagen, which induces cancer cell metastasis via promoting the primary tumour to migrate. 37 Meanwhile, CXCR4 was reported to be a marker protein of metastasis in gastric cancer cells.^38^ In this study, we firstly proved that CBX4 knockdown significantly inhibited the wound closure in scratch migration assay and cell invasion in transwell assay (Figure 5A‐D). In addition, the expressions of MMP2, MMP9 and CXCR4 decreased in CBX4 knockdown cells, indicating the poor metastases (Figure 5E). On the other hand, cancer cell invasion and metastasis are highly linked with the presence of filopodia.^39^ In breast cancer, increased formation of filopodia was found to strengthen the migration of cells, and cell migration in vitro and metastasis in vivo were suppressed by the inhibition of filopodium formation.^30^, ^31^ We observed that filopodium formation in lung cancer cells was inhibited by CBX4 knockdown, suggesting the attenuation of metastatic abilities (Figure 5F).

CBX4 is able to regulate the expression of BMI‐1 in the DNA damage response.^16^ We hypothesized that CBX4 may regulate the expression of BMI‐1 to increase tumour growth and metastasis. The expression of BMI‐1 and P38 was controlled by mitogen‐activated protein kinase kinase 3(MKK3) in hepatocarcinogenesis via cell cycle control mechanism of hepatocellular carcinoma cells (HCC) and inhibition of cell G1‐S transition and proliferation.^40^ Similarly, BMI‐1 was inhibited by miR‐203 in myeloma cells via regulating G1/S transition.^41^ Meanwhile, the expression of MMP2 and MMP9 via the PTEN/ pI3K/AKT pathway was increased by BMI‐1 overexpression in HCC tissue and cells contributing to invasion and metastasis.^42^ The results in this study show a close association between the expression of CBX4 and BMI‐1. Firstly, our Western blot analysis data showed that CBX4 knockdown decreased the expression of BMI‐1; however, overexpression of BMI‐1 did not affect the expression of CBX4 (Figure 6A). Secondly, CBX4 knockdown decreased colony formation and cell invasion, whereas BMI‐1 overexpression attenuated shCBX4‐inhibited colony formation and cell invasion (Figure 6B,C). Thirdly, CBX4 knockdown decreased the expression of cell cycle‐related proteins (P53, CDK2 and Cyclin E) and cell migration‐related proteins (MMP2, MMP9 and CXCR4), while BMI‐1 overexpression reversed the down‐regulation of P53, CDK2, Cyclin E, MMP2, MMP9 and CXCR4 induced by CBX4 knockdown (Figure 6D,E). These results indicate that CBX4 is an upstream protein of BMI‐1 which regulates the proliferation and migration in lung cancer cells through BMI‐1.

In vivo, similar results were obtained which showed that tumour sizes and the expression of Ki67 were all regulated by CBX4 (Figure 7A‐E). Furthermore, knockdown of CBX4 resulted in less metastatic nodules in the livers compared to the control group (Figure 7F). The above results indicated that CBX4 might be a potential curable target of proliferation and metastasis in lung cancer.

In summary, our study demonstrates a mechanism by which CBX4 enhances the proliferation and metastasis of lung cancer in vitro via up‐regulating the expression of BMI‐1, thereby increasing the expression and activity of P53, CDK2, Cyclin E, MMP2, MMP9 and CXCR4. Our findings indicate that CBX4 is a promising target that may be used as a biomarker for early diagnosis and that inhibition of CBX4 could be a potential therapeutic target to inhibit the proliferation and metastasis in lung cancer.

## CONFLICT OF INTEREST

All authors have no conflicts of interest.

## AUTHORS’ CONTRIBUTIONS

CPH, QZ and GBL conceived and designed the study and wrote the manuscript. CPH and QZ performed the experiments. QT, HYZ and WYL collected the data. JBH, YLL and QW analysed the data. HYZ, JZ, MZ, FFS, JT and WJL interpreted the data. GBL and RZ reviewed the manuscript. All authors read and approved the final version of the manuscript.

## Data Availability

All the datasets generated and analysed are available from the corresponding author on reasonable request.
